# MWNTs or PEG as Stability Enhancers for DNA–Cationic Surfactant Gel Particles

**DOI:** 10.3390/ijms22168801

**Published:** 2021-08-16

**Authors:** Amalia Mezei, Ramon Pons

**Affiliations:** Departament de Tensioactius i Nanobiotecnologia, Institut de Química Avançada de Catalunya, IQAC-CSIC, C/Jordi Girona 18-26, 08034 Barcelona, Spain; amalia.ullrichmezei@jci.com

**Keywords:** DNA, multi-walled carbon nanotubes, PEG, composite, release, stability, SAXS

## Abstract

Cationic surfactants interact with DNA (Deoxyribonucleic acid), forming surfactant-DNA complexes that offer particularly efficient control for encapsulation and release of DNA from DNA gel particles. In the present work, DNA-based particles were prepared using CTAB (Cetyltrimethylammonium bromide) as the cationic surfactant and modified using two different additives: (Multi-Walled Carbon Nanotubes) MWNT or PEG (Poly Ethylene Glycol). The use of both additives to form composites increased the stability of the gel particles. The stability was monitored by the release of DNA and CTAB in different pH solutions. However, not much is known about the influence of pH on DNA–surfactant interaction and the release of DNA and surfactant from gel particles. It was observed that the solubilization of DNA occurs only in very acid media, while that of CTAB does not depend on pH and gets to a plateau after about 8 h. Within 2 h in contact with a pH = 2 solution, about 1% DNA and CTAB was released. Complete destruction for the gel particles was observed in pH = 2 solution after 17 days for PEG and 20 days for MWNT. The composite particles show a considerably enlarged sustained release span compared to the unmodified ones. The dehydration-rehydration studies show that the structure of the composite gel particles, as determined from SAXS (Small-Angle-X-Ray-Scattering) experiments, is similar to that of the unmodified ones. These studies will allow a better knowledge of these particles’ formation and evolution in view of possible applications in drug delivery and release.

## 1. Introduction

Cationic surfactants interact with DNA, forming surfactant-DNA complexes [1]. These complexes can be produced in the form of core-shell gel particles formed by the coprecipitation of DNA and cationic surfactant entropically driven by the release of counterions to the solution [2,3,4]. The main applications envisaged for these complexes are gene delivery and encapsulation. A clear understanding of the interaction between DNA and cationic surfactant and, in particular, the stability of the gel particles has prompted us to study DNA-based gel particles. As the DNA-based gel particles that have a high DNA content are promising DNA vehicles for use in non-viral gene delivery systems. For this kind of vehicle, the stability at different pHs is a key factor. In particular, the possible use of these particles as oral delivery vehicles prompted us to improve the stability in acidic media.

The development of non-viral biocompatible vectors for efficient intracellular transfection of nucleic acids, such as DNA and RNA, is one of the most critical challenges toward gene therapy. Early studies showed that functionalized carbon nanotubes were able to bind DNA plasmids for gene transfection [5,6]. Despite concerns about the toxicity of carbon nanotubes, the advantages of their use and a better control of their properties could outbalance the risks and mitigate the possible toxicity [7,8]. In the last decades, it was observed that the functionalization of fullerenes with biological and bioactive molecules such as proteins, carbohydrates and DNA, for example, had a tremendous impact on the design of much needed drug-delivery systems, biosensors and other biological devices [9,10,11,12]. The application of carbon nanotubes in biomedical applications has already shown significant promise [13,14,15,16,17]. Such applications include their use as gene carriers, protein and DNA biosensors or ion channel blockers [18,19,20,21]. Multi-walled carbon nanotubes (MWNT) have been used previously as drug delivery vehicles [14,22]. They have also been studied for drug delivery applications in pH-sensitive systems [23]. Moreover, it has been shown that a pH shift from 7.2–7.4 in the blood or extracellular spaces to 4.0–6.5 in the various intracellular compartments, which takes place during cellular uptake, can be used for intracellular drug delivery [24]. The biocomplexes of neutral molecules and DNA are also potential candidates for clinical applications, including biosensors and gene and drug delivery system development. The stability of gel particles formed by the interaction between neutral molecules and DNA plays an important role in the field of medicinal and pharmaceutical applications. The calf thymus DNA and polyethylene glycol (PEG) interaction was earlier studied by different methods and as a function of pH [25]. The results showed that PEG stabilized the DNA structure and that the interaction is weak to moderately strong. Additionally, PEG is a well-studied and widely used polymer not only to synthesize a variety of linear copolymers but also to improve the processability, water solubility, and prolonged blood circulation of carbon nanotubes through non-covalent interactions [26]. Therefore, PEG is a prototype of an inert, biocompatible polymer. The inertness and non-toxic properties of PEG give rise to a number of applications as a non-ionic surfactant and as an additive in cosmetics, pharmaceuticals, and food. PEG has been used in many drug delivery systems because of its high solubility in water and improved biocompatibility. In gene delivery systems, PEG has also been coupled to polycationic polymers or liposomes to improve solubility of complexes or transfection efficiency [27,28]. PEG usually is used in contact-lens fluid, detergents and lotions, as an adhesive, as a thickener in acid cleaners and for drag reduction, foam stabilization, lubrication and oil-well flooding applications in industries, cosmetics, drug or pharmaceuticals.

On the other side, the third crucial component of the present gel particles are the cationic surfactants, which are active agents with antibacterial, antistatic, dispersants, emulsifying, wetting and solubilizing properties [29,30,31]. The cationic surfactants have also been tested for gene delivery and gene transfection, which have been involved in gene therapy [32,33]. Many cationic surfactants also showed interesting properties for antibacterial applications and complex formation. Cetyltrimethylammonium bromide (CTAB) was used to control the rate of DNA denaturation and to precipitate selectively plasmid DNA from RNA proteins and endotoxins [34]. Surfactants self-assemble in water or aqueous solutions forming micelles. The critical micellar concentration (CMC) for CTAB is of 0.98 mM according to Okuda et al. [35]. Ionic strength and common ion effects strongly reduce this value, for instance, to 0.14 mM in the presence of NaBr.

In the present work, the DNA–CTAB gel particles have been modified by including MWNT or PEG into the formulation of DNA in order to increase their stability. The stability was monitored by the determination of the release of DNA and CTAB from the gel particles in different pH conditions, and we compared the results with unmodified DNA–CTAB gel particles. The presence of additives increases the stability of the particles and also changes the release pattern. In addition, the structural changes in the presence of MWNT and PEG were also studied. The complexes only suffer minor structural modifications in the presence of the additives. The modified particles can be dehydrated and rehydrated keeping the structure and barrier function. Those particles could be used as formulations for drug delivery through the stomach, where they would suffer some degeneration at pH = 2 (about 1% material within 2 h) but would preserve the integrity, leading to possible delivery in the gut. The possibility of using this for gene delivery does not seem too reasonable. Although, due to their size, the present particles cannot be used as vehicles for systemic use (as formulations for drug delivery or for gene delivery), the information gathered here concerning the stability and formation may be useful for envisaging nanoparticles for this purpose.

## 2. Results

**Figure 1:**
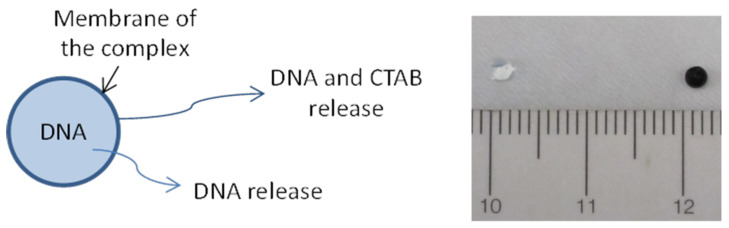
Sketch of the studied delivery systems and, on the right side, a picture of PEG–DNA–CTAB and MWNT–DNA–CTAB gel particles, which were for two hours in the CTAB solution. The size of the gel particles was the same, as the DNA gel was added drop-wise to the CTAB solution.

The stability of gel particles formed by interaction of neutral molecules, DNA and cationic surfactant has an important role in different applications, such as pharmaceuticals, cosmetics and gene therapy. The DNA during the formation process is enclosed inside the gel particle and the membrane is formed by the co-precipitation of DNA and cationic surfactant. The gel particles as viable delivery systems can be schematically described, as shown in Figure 1. The DNA is encapsulated in the gel particles, and the complex of MWNT–DNA–CTAB and PEG–DNA–CTAB form the permeable membrane. In the preparation process of gel particles, the particles are in contact with the CTAB solution for a limited time; therefore, it is important to know the kinetics of membrane formation to better understand the characteristics of the particles. For the possible uses of these particles, it is important to know their stability in different solutions. The stability of the membrane is related to the release of its constituent molecules. In addition, the process of drying-rehydration, useful for long term storage, will depend on the exact gel particle structure and composition.

### 2.1. Kinetics of Gel Particle Formation

**Figure 2:**
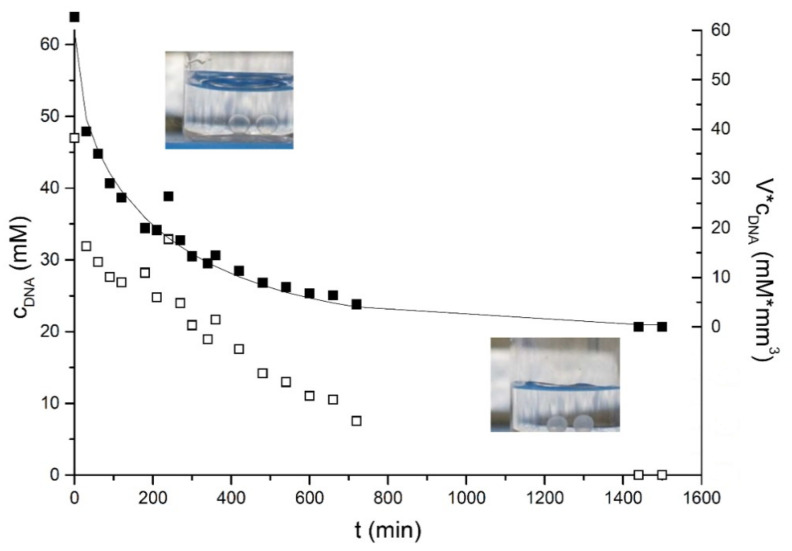
DNA concentration inside of the gel particles as a function of time and amount of DNA in the gel particles core during the formation process. The open symbols correspond to the concentration scale and the full symbols to the V*c_DNA_ (Total DNA) scale. The inset images show the visual change of the gel particles. The line corresponds to the best fit for Equation (1).

In order to understand the kinetics of gel particles formation, we have followed the process of contacting a droplet of concentrated DNA solution with excess CTAB solution over a period of 30 h. From a visual point of view, with time, the gel particles membrane was getting thicker and whiter. In Figure 2, the changes in the DNA concentration inside the gel particles over time are shown. The inset photos in the figure show the appearance changes of the gel particle; in the left image, the gel particles are transparent, and in the right image, their membrane is white. The concentration of DNA decreases regularly with time after a sharp decrease corresponding to the first 30 min. The odd point at 4 h could be related to a restructuration of the complex membrane; at this time, a second precipitate was observed inside of the particle. This double membrane structure was more evident after 24 h.

As we can see, the DNA concentration in the gel particle decreases with time. The decrease in the first hour is faster and later slows down. After 24 h, the concentration of DNA in the gel particle was at the detection limit. The diffusion of DNA inside of the gel particles can be described using Fick’s law of diffusion. Considering the spherical geometry of the gel particles and the boundary conditions: (a) the constant initial concentration of DNA inside the particles at time zero and (b) zero DNA activity at the particle boundary, the diffusion can be solved. Furthermore, there is no DNA excess in the bulk: the DNA concentration inside of the gel particles decreases with time, and the membrane thickness of the gel particles increases. This corresponds to the mode of diffusion for a “monolithic system” in spherical form [36]:

(1) $$\frac{M_{rt}}{M_{\infty }}=\frac{6}{\pi^{2}}\sum_{n=1}^{\infty }\frac{exp\left(-Dn^{2}\pi^{2}\frac{t}{R^{2}}\right)}{n^{2}},$$

where *M_rt_* and *M_∞_* denote the remaining amounts of DNA in the core at time *t* and the amount released at infinity, respectively; *R* is the radius of the sphere; *D* is the diffusion coefficient of DNA.

To obtain the total amount of free DNA inside the gel particles core, the core volume has to be known. To obtain this value, the radii of the gel particles and the thickness of the membrane were measured at different times. The volume inside the particles was fitted to an exponential decay to obtain interpolated values at times where the membrane thickness had not been measured. For example, the values obtained experimentally for the membrane thickness after 2 h is 0.1 mm, and after 24 h, the membrane was 0.2 mm thick for the total radii of 1 mm. Then, the total amount of free DNA corresponds to the measured concentration times the core volume. It should be noted that in Equation (1), the radius of the core of the particle is also a function of time. The fit of Equation (1) in Figure 2 results in the diffusion coefficient of 3.5 ± 0.5 × 10^−12^ m^2^/s. This value should be compared to that calculated from the DNA molecular weight of 1300 kDa and the semiempirical equation of Lukacs et al. [37] *D* = 2 × 10^−12^ m^2^/s. In this simple model, we have also assumed that as soon as a DNA molecule reaches the inner part of the membrane, it precipitates. This implies that the diffusion of CTAB through the membrane is fast compared with the diffusion of DNA within the gel particle core, which seems a reasonable assumption. Additionally, the diffusion of CTAB inside the particle has not been considered. This diffusion seems to occur, giving rise to the inner precipitate observed after about 4 h that at latter times forms a secondary membrane. The rationale for the formation of this secondary inner membrane relies on the different diffusivities of DNA and CTAB and the concentration of both compounds needed for precipitation. While at the initial stages, both DNA and CTAB are well above the threshold for coprecipitation, and after some time, the concentration of DNA in the core of the particle must show a gradient. The faster counter diffusion of CTAB seems to be enough to form a second front of coprecipitation. This further decrease in free DNA could explain the slightly faster diffusion coefficient measured.

In the process of preparing the gel particles to study the release, the particles are in contact with the CTAB solution for 2 h. Thus, there is a significant amount of free DNA in the core of these gel particles, which has an assumed nearly uniform concentration. The release of DNA and CTAB from this type of system has been studied at a fixed pH by keeping the gel particles under constant agitation in a set pH solution. DNA can be released from the core of the particle but also from the membrane, and the CTAB is released only from the membrane (except for some low concentration below the precipitation threshold), as it is part of the complex.

### 2.2. DNA Release in Different pH Solution

**Figure 3:**
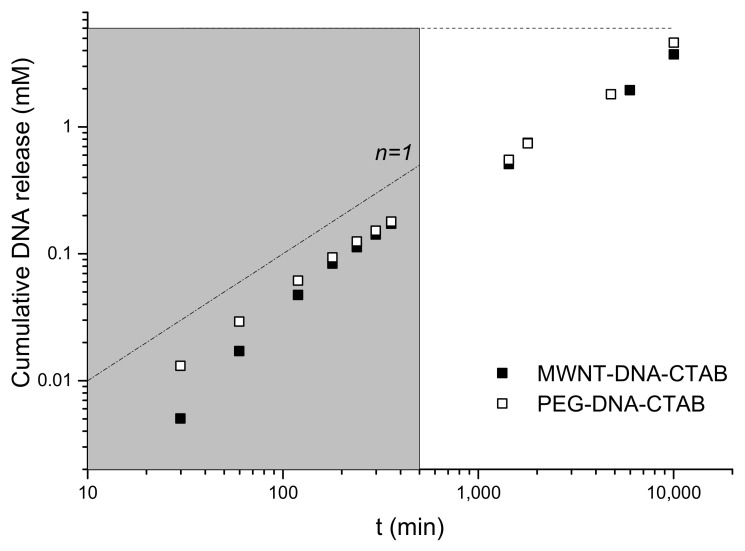
Cumulative released DNA concentration as a function of time in pH = 2 solutions. The dashed line corresponds to the maximum DNA concentration what can be released. The gray area shows the fast release region.

The DNA release of MWNT–DNA–CTAB and PEG–DNA–CTAB gel particles was followed in different pH solution. Significant release was observed only in acid media, at higher pH the released amount of DNA was below the limit of detection of our method. In Figure 3, the total released DNA is shown as a function of time at pH = 2 for MWNT–DNA–CTAB and PEG–DNA–CTAB gel particles. The release for the two types of gel particles shows similar behavior: in the first hours, it is fast (gray region), where the slope for the PEG–DNA–CTAB gel particles is 1 (*n* = 1), and after some time, the release speed decreases. The MWNT–DNA–CTAB gel particles initially showed a faster release, which may be due to some DNA loosely attached to the surface of the particles. The release in the later stage, although slower, can be considered as a sustained release (note the doubly logarithmic scale). The amounts of released DNA in solutions of pH = 7 and pH = 9 were around the detection limit of the spectrometer and are not shown in the graphic.

If we assume the conditions, which are formally the same for reservoir systems with non-constant activity source [36], that the gel particles membrane influence the release process and the DNA release from gel particles can slow by passing through the membrane. Under these conditions, the following equation can be derived:

(2) $$\frac{M_{t}}{M_{\infty }}=1-exp\left(-\frac{ADKt}{VL}\right),$$

where *M_t_* and *M_∞_* denote the cumulative amounts of DNA at time *t* and infinity, respectively; *A* is the total surface area of the device (*A* = 4πR^2^, R the radius of the particle); *D* is the diffusion coefficient of DNA within the membrane; V is the volume of the gel particle (V = 4/3πR^3^); *K* is the partition coefficient of DNA between the membrane and the reservoir; and L is the thickness of the membrane (*R*−*R_i_*) [38].

(3) $$\frac{M_{t}}{M_{\infty }}=1-exp\left(-\frac{3RDKt}{R_{i}^{2}R-R_{i}^{3}}\right),$$

The fitting of Equation (3) to the data in Figure 3 produces diffusion coefficients of 3 × 10^−14^ m^2^/s for *R* = 1 mm and *R_i_* = 0.9 mm. This value considers the partition coefficient *k* = 1 and is about two orders of magnitude smaller than the diffusion coefficient of DNA in aqueous solution. Because this process takes part in acidic media, this mean value may not correspond strictly to the diffusion of DNA through the membrane but also to the destruction of the membrane itself due to the degradation of DNA. This value can be compared with the release of DNA from non-modified gel particles, which results in a diffusion coefficient of 7 × 10^−13^ m^2^/s (applying Equation (3) to the data of reference [39]).

**Figure 4:**
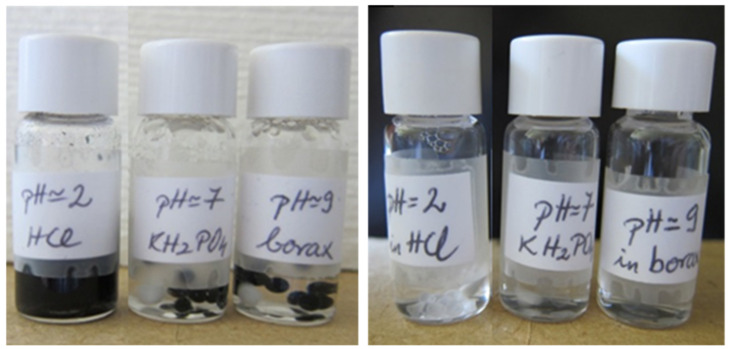
MWNT–DNA–CTAB and PEG–DNA–CTAB gel particles in different pH solution after one week.

As it can be seen, the released amount of DNA after one week (~10,000 min) at pH = 2 is around 62% for MWNT–DNA–CTAB and 76% for PEG–DNA–CTAB complexes and after 17 days is complete, the gel particles are dissolved in the solution. For the MWNT–DNA–CTAB particles, after 10 days in pH = 2 solutions, it was observed that a visible amount of carbon nanotubes at the bottom of the vials with gel particles were still present. After 20 days, the gel particles were dissolved/disaggregated, and pieces of membrane were still dispersed in the solution. The PEG–DNA–CTAB gel particles were more stable in the pH = 2 solution than the MWNT–DNA–CTAB gel particles, but they stuck together and, after 17 days, were completely dissolved. This behavior was compared to that of unmodified gel particles, which were solubilized after 14 days in the same conditions. In Figure 4, images of the particles in different solutions are shown after one week.

After one month, the particles were still stable in pH = 7 and pH = 9 solutions. Measurements of the DNA concentration in the outer solution and inside the particles were carried out. The latter were performed by opening one gel particle from each solution to determine the DNA and CTAB concentrations in the liquid core of the particles. The DNA concentrations inside the particles were MWNT–DNA–CTAB >> DNA–CTAB > PEG–DNA–CTAB, and the concentration in the supernatant solutions was approximately the same. Table 1 shows the results for one-month-old samples. These differences in “free” DNA in the core of the particles cannot be explained by differences in particle size but should be a reflection of the formation of additional precipitate produced by changes in the complex structure. The concentration of CTAB found in the interior of the particles is notable because this is well above the surfactant CMC (which is around 1 mM in water and lower at higher ionic strength). It is plausible that the transfer of CTAB is triggered by the formation of some conjugation of the DNA with the CTAB. Moreover, according to Dias et al. [40], at this DNA/CTAB concentration, two-phase systems should form, but in the core of the particles, no precipitation was observed; these solutions were viscous but transparent. A possible explanation for this finding would be related to the small size of the core of the particles combined with the absence of shearing forces, allowing for a strong oversaturation. The released amount of DNA and CTAB after one month is still very small at pH = 9 and slightly bigger than in pH = 7.

### 2.3. CTAB Release in Different pH Solution

**Figure 5:**
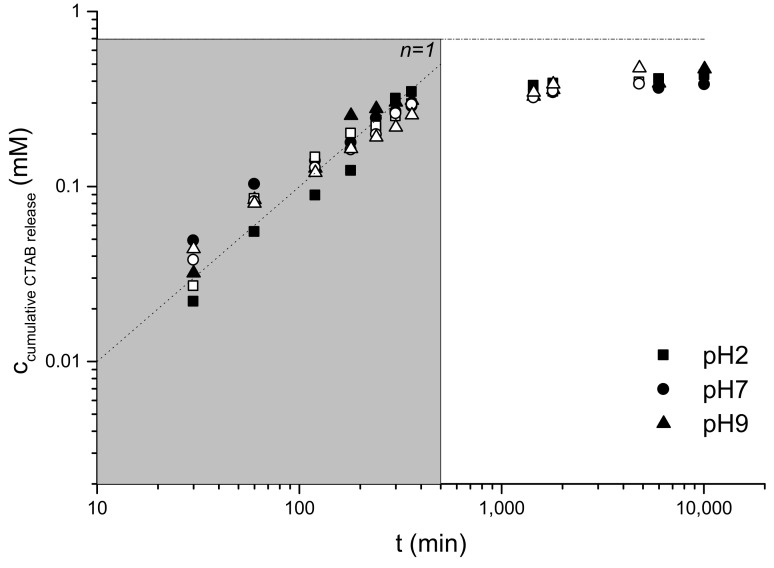
The cumulative released surfactant concentration as a function of time in the different pH solutions for MWNT–DNA–CTAB gel particles (solid symbols) and for PEG–DNA–CTAB gel particles (open symbols). The gray area shows the fast release region. The dashed line in the figure corresponds to the maximum released amount of surfactant.

The cumulative release of the surfactant in different pH solutions is presented in Figure 5 for MWNT–DNA–CTAB (solid symbols) and PEG–DNA–CTAB (open symbols). The release of surfactant is fast in the first hours, and after 7 h, the release rate is severely reduced. As we can see at pH = 2, 7 and 9, the released surfactant amount is similar. The fast release part can be described by a normal diffusion (slope 1 in log-log plots) and after 7 h as the release corresponds to a slow diffusion regime. This change can be explained by the release from the membrane in the first hours and the release from the core and membrane of the gel particle in the slow-release regime. Additionally, a change in the mechanism from monomer to micelle could be associated to this change since it occurs when the concentration reaches a value in the range of CTAB CMC at those ionic strengths. At pH = 2, a complete release was observed after 17 days, when the particles were completely dissolved. At a higher pH, as the particles are not diluted/degraded, total solubilization was not observed. Even after one month, the particles at higher pH solutions were stable.

The release in PEG–DNA–CTAB gel particles is slower than in MWNT–DNA–CTAB but still fast up to 400 min and then slows down. The release studies showed that, in the first hours, there are differences in CTAB release, but after 400 min, the release is similar in the different solutions and particles. With increasing pH, the release is slower in the first hours. The released amount of CTAB after one week (~10,000 min) is around 68% for MWNT–DNA–CTAB and PEG–DNA–CTAB complexes. Additionally, it was observed that the particles are stable for up to 17 days, and the release was more controlled. This observation agrees with earlier results, which indicated that MWNTs can stabilize hydrogels; a slower swelling is recorded because of smaller diffusion coefficients of solvent in the polymeric network [41]. The effect of PEG and MWNT on the structure of the hexagonal liquid crystalline phase is small (see below); hence, the mechanism of stabilization of this phase with respect to the solubilization does not seem to rely on the modification of the microstructure. There is a non-solved question concerning the structure of these particles referring to the tridimensional disposition of the hexagonal liquid crystalline structure on the surface of the particle. Those particles are big enough for the shell to be considered effectively flat with respect to the dimensions involved in the tridimensional arrangement of CTAB micelles and DNA chains. Then, the question remains whether the axis of the rods forming the liquid crystal are preferentially oriented perpendicular to the local plane of the shell or along its director vector or it just corresponds to a mosaic of very small crystals without a preferential orientation. In the latter case, it is clear that the process of solubilization in the acidic media will preferentially attack the grain boundaries. The role of PEG or MWNT would be to avoid the crumbling of the structure by intimately linking a number of grains and constituting an effective network. If there is a preferential direction of the rods, the whole shell could be a single crystal with some point or line defects [42] or still a mosaic, in which case, the mechanism of stabilization would be parallel to that explained before, that is, holding together different grains. The fitting of Equation (3) produces diffusion coefficients for CTAB of 1.48 ± 0.15 × 10^−12^ m^2^/s for PEG and 1.89 ± 0.27 × 10^−12^ m^2^/s for MWNT. These values are about 100 times smaller than the diffusion coefficient for CTAB in water. This coincidence in the reduction of the apparent diffusion coefficient for both the surfactant and DNA when released from the gel particles compared to the diffusion coefficients in water may imply that the processes by which they are released are similar. That is, the apparent obstruction effect in the membrane is similar for both components of the membrane.

### 2.4. Dehydration and Hydration of the Gel Particles

The first observation for the modified DNA gel particles was the visual aspect. The size of the two modified gel particles was similar, around 1–2 mm (as it can be seen in Figure 1), but the weight was quite different. The PEG–DNA–CTAB (white) particle was 9.5 mg and the MWNT–DNA–CTAB (black) particle 6.7 mg. This difference corresponds to the small size difference in the gel particles (1.31 mm and 1.17 mm radii, respectively).

The particles were stable in water, in 10 mM NaBr and high pH solution at room temperature. Gel particles prepared and washed in the usual way were dried under vacuum at room temperature. The dried gel particles were inserted in water, and the rehydration was followed. When the dry particles were immersed into aqueous solution, the recovery of the original shape depended on the contact time of the gel particles with the CTAB solutions during the preparation process. Gel particles prepared by keeping them two hour or less in CTAB solution recovered the shape completely. Slightly longer times resulted in partial recovery, while particles kept in contact with CTAB for more than 6 h did not show appreciable swelling of their cores. Recalling the results shown in Section 2.1, we can see that when the “free” DNA concentration was above 30 mM, the particles rehydrate completely. Decreasing the concentration of “free” DNA produces only partial recovery, and under 10 mM of DNA, the rehydration is very small. In this latter case, only the membrane of the gel particle is hydrated, but they do not keep the original form. From these observations, we can conclude that the rehydration process is driven by the osmotic pressure of the “free” DNA encapsulated in the core of the particles, with some contribution also of the PEG in the case of the PEG modified particles.

The hydration process has been followed in more detail for gel particles prepared after 2 h contact with CTAB solution. The hydration of the particles is slow, but they rehydrate completely [39,43]. The structure of the modified DNA gel particles by SAXS measurements does not show differences. In the dry state, the repeating distance is around 40 Å and 48 Å in the fully hydrated form. The repeating distances of the dry and rehydrated gel particles compared with DNA–CTAB gel particles are presented in Table 2.

**Figure 6:**
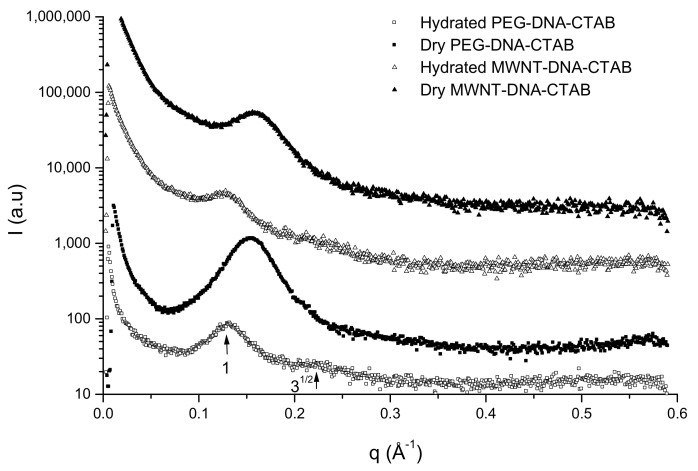
SAXS spectra of the dry (solid symbols) and hydrated (open symbols) gel particles. The arrows show the first and the second peaks of the hexagonal packing. The spectra are scaled for clarity.

**Figure 7:**
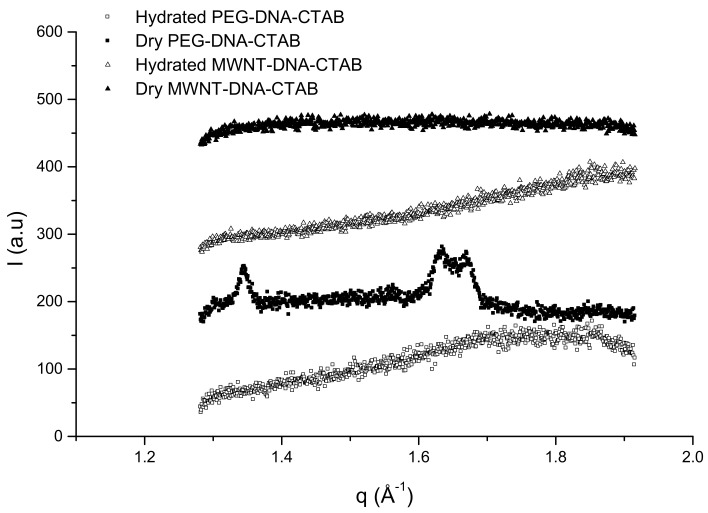
WAXS spectra of the dry (solid symbols) and hydrated (open symbols) gel particles at room temperature. The spectra are scaled for clarity.

The modified DNA gel particles in the dry state show the same structure as DNA–CTAB, a very close packed structure, but when they are hydrated, the repeating distance is smaller with around 1–2 Å compared to the DNA–CTAB complex. This difference shows that in the presence of MWNT and the PEG, the DNA is more compacted. The SAXS spectra of the dry and hydrated gel particles are shown in Figure 6, and the WAXS spectra can be seen in Figure 7.

The repeating distance increases with time as the particles are getting hydrated and is constant after 4 h [44]. The hydrated form of the gel particles shows a hexagonal structure, which is best defined for PEG–DNA–CTAB gel particles (the arrows in Figure 6 indicate this structure). The WAXS spectra of the dry PEG–DNA–CTAB gel particles show an ordered structure, which can be due to the possible interaction between PEG molecules and DNA segments. Additionally, the peaks in the WAXS profile corresponds to the two main characteristic peaks of PEG crystals at 25 °C at *q* = 1.35–1.63 Å^−1^ [44,45].

The results of X-ray measurements suggest that the structure of the DNA–CTAB complex is only slightly modified in the presence of MWNT or PEG. From the results, we can conclude that the particles after drying can be hydrated keeping the core-shell form, structure and function. The hydrated core-shell particles may be used as drug delivery systems and can give a basis for developing DNA-based carriers.

## 3. Materials and Methods

### 3.1. Materials

The sodium salt of salmon testes deoxyribonucleic acid (DNA with a mean molecular weight of 1300 kDa, which corresponds to 2000 base pairs and an approximate length of 660 nm), sodium bromide (NaBr), hydrochloric acid (HCl) (for pH = 2 solution), borax (for pH = 9 solution) and poly(ethylene glycol) 10,000 (PEG) were from SIGMA. Potassium phosphate monobasic (for pH = 7 solution) and CTAB were from FLUKA. CMC of CTAB at 25 °C is 0.98 mM in water and 0.14 mM in the presence of 10 mM NaBr, according to Okuda et al. [35]. The multi-walled carbon nanotubes (MWNT) were from Nanostructured & Amorphous Materials Inc. (Houston, TX, USA). The used MWNTs were 94% pure, stock No. 1240XH and with an outer average diameter between 20 and 30 nm and length between 0.5 and 2 μm. Deionized water was used for the preparation of the diverse solutions.

### 3.2. Gel Particle Preparation

For the gel particle preparation, different solutions were used, namely: 10 mM NaBr, 2% DNA, 2% CTAB, 0.5% MWNT and 1% PEG. In the process of preparation, a droplet of DNA solution is introduced in the surfactant solution. The droplet volume controls the size of the gel particle produced. We used particles from 6.7 to 9.5 mg with radii spanning from 1.2 to 1.3 mm. Internal volume and membrane thickness were obtained by weighing individual particles, cutting them, weighing the remaining membrane and measuring their thickness.

#### 3.2.1. DNA–CTAB Gel Particles

First, the DNA solution was prepared using 10 mM NaBr. The DNA solution was added drop-wise into the CTAB solution, and the formed gel particles were kept under stirring. The DNA diffusion inside of the gel particle was followed with time, first after half an hour, and later every hour, one gel particle was washed with deionized water (10 × 3 mL) to remove the excess CTAB and opened, then the DNA solution removed and the membrane thickness measured.

#### 3.2.2. MWNT–DNA–CTAB Gel Particles

First, the MWNTs were sonicated in 10 mM NaBr solution and, after that, mixed with DNA. The DNA–MWNT solution was added drop-wise into the CTAB solution, and the formed gel particles were kept under stirring.

#### 3.2.3. PEG–DNA–CTAB Gel Particles

First, the PEG solution was prepared in 10 mM NaBr and, after that, mixed with DNA. The PEG–DNA solution was added drop-wise into the CTAB solution, and the formed gel particles were kept under stirring.

#### 3.2.4. Release Studies

After two hours, the modified gel particles were washed with deionized water (10 × 3 mL) to remove the excess CTAB, MWNT–DNA and PEG–DNA solution and a few particles were suspended in different pH solutions (1 mL). The release was followed with time, first after half an hour, and later every hour, the supernatant solution was changed, and the particles were re-inserted in fresh solution. The samples during the release studies were agitated and kept above CTAB Krafft temperature, 27 ± 0.5 °C.

### 3.3. pH Measurements

pH measurements were carried out with a Thermo Scientific Orion Dual Star pH meter and an Orion 8103SC, ROSS Semi-Micro Comb pH electrode (Thermo Fisher Scientifics Inc., Beverly, MA, United States) at room temperature.

### 3.4. Surfactant Concentration Determination

To determine the surfactant concentration, the surface tension of a determined dilution is measured as well that dilutions from the starting concentration. The concentration is determined from the superposition of this curve with the reference curve of known CTAB concentration.

The surface tension measurements with a homemade pendant drop instrument. [46,47] The droplet of the solution was formed at the end of a straight-cut Teflon tube, which had an internal diameter of 0.8 mm and an external diameter of 1.58 mm. The image of the droplet was filmed using a webcam (640 × 480 pixels) and corrected for spherical aberration. The droplet contour was taken at the point of maximum slope of the intensity and was fitted to the Laplace–Young equation using a homemade golden section search algorithm. [48] Water was put in the droplet chamber to prevent evaporation.

### 3.5. DNA Concentration Determination

The DNA concentration was determined by measuring the absorbance at 260 nm with a UV-VIS spectrophotometer CARRY 300 (Agilent, Santa Clara, CA, United States). Because the absorbance depends on pH, calibration curves were measured at each pH.

### 3.6. SAXS-WAXS Determination of the Composite Nanostructure

X-ray measurement: Small and wide-angle X-ray (SAXS and WAXS) measurements were carried out using an S3-MICRO (Hecus X-ray systems GMBH, Graz, Austria) coupled to a GENIX-Fox 3D X-ray source (Xenocs, Grenoble, France), which provides a detector focused X-ray beam with the Cu K_α_ line (1.542 Å) with more than 97% purity and less than 0.3% K_β_. Transmitted scattering was detected using a PSD 50 Hecus with a pixel resolution of 54.2 μm and approximately 1 cm pixel width. The samples were inserted in a capillary with a 1 mm diameter. The SAXS scattering spectra are shown as a function of the scattered vector modulus q according to:

q = 4π/λ sin (ϴ/2),

where λ is the wavelength of the used X-ray (1.542 Å), and ϴ is the scattering angle. The scattering patterns are shown as obtained, which is mainly with the detector smearing.

The samples for X-ray were prepared by vacuum drying the particles and introducing them in a 2 mm diameter glass X-ray capillary (10 μm wall thickness, Hilgenberg GmbH, Malsfeld, Germany). The rehydration of the samples was performed in the same capillary by adding excess water.

### 3.7. Photography

The images presented in this article were acquired with a Canon PowerShot S90 Wide Zoom (Canon, Tokyo, Japan) digital camera.

## 4. Conclusions

The formation of gel particles was followed by measuring the DNA concentration in the core of the particles as a function of time. The picture agrees with the membrane forming by coprecipitation of DNA and CTAB at the droplet surface, and the kinetics agree with a DNA diffusion limited model. The modified MWNT–DNA–CTAB and PEG–DNA–CTAB gel particles were studied in different pH solutions. The composite gel particles have shown higher stability in the presence of encapsulated multi-walled carbon nanotubes compared to the pristine DNA–CTAB capsules. The results show that the modified gel particles membrane was stable in acid media for 20 and 17 days, respectively, compared to 14 days for the unmodified particles. After 7 days, the release of DNA and surfactant reach values close. Additionally, the release of DNA in acid media was slower and more controlled, i.e., after 7 days, 62% of DNA was released from MWNT–DNA–CTAB compared to 76% from PEG–DNA–CTAB. The gel particles only release the DNA in very acidic solutions (pH = 2), while at a higher pH, the release is marginal. In milder acidic conditions, the particles show higher stabilities after one month. The increased stability and modulated release is compatible with either PEG or MWNT linking and avoiding crumbling of the different liquid crystalline domains of the shell. It was observed that the CTAB release does not depend on the pH of the storage solution. The released amount of CTAB from the studied gel particles after one week is around 68% of the total. SAXS measurements of dried particles show that the modified gel particles have the same structure than that observed in DNA–CTAB particles. However, in the hydrated state, shorter distances are obtained for the modified particles than for the non-modified ones, showing the signals of close packed hexagonal structure for all of them. For PEG–DNA–CTAB gel particles, this hexagonal structure is more defined than for MWNT–DNA–CTAB particles. The studied gel particles show potential behavior for pharmaceutical application and a controlled DNA encapsulation. The new carriers are undoubtedly attractive and deserve further investigation.

## Figures and Tables

**Table 1:** DNA and surfactant concentrations inside and outside of the gel particles.

Sample	C_DNA_ (mM)		C_CTAB_ (mM)	
	C_inside_ (mM) ^1^	C_outside_ (mM)	C_inside_ (mM)	C_outside_ (mM)
WNT–DNA–CTAB pH = 7, KH_2_PO_4_	29	0.008	3.8	0.023
WNT–DNA–CTAB pH = 9, borax	27	0.029	5.4	0.20
PEG–DNA–CTABpH = 7, KH_2_PO_4_	10	0.033	3.8	0.033
PEG–DNA–CTABpH = 9, borax	14	0.076	5.0	0.14
DNA–CTAB 10 mM NaBr	17	0.001	3.8	0.002

^1^ the salmon DNA concentration inside of the gel particle after 2 h in the CTAB solution and washing with water is around 35.94 mM (initial DNA concentration inside the gel particles).

**Table 2:** Repeating distances of the dry and rehydrated gel particles at 25 °C.

Sample	MWNT–DNA–CTAB	PEG–DNA–CTAB	DNA–CTAB
Dry particle	39.52 Å	40.54 Å	40.80 Å
Hydrated particle	48.71 Å	47.96 Å	50.27 Å

## Data Availability

Data is contained within the article.
